# Death of Dr. Caldwell, of Atlanta

**Published:** 1886-03-20

**Authors:** 


DEATH OF DR. CALDWELL, OF ATLANTA.—Dr. J. J. Caldwell, of our city, died on Sunday last—aged sixty-six years—and was buried on Tuesday, the 16th instant. The physicians of the city attended his funeral, officiated as pallbearers, and evinced marked sympathy and respect for their departed brother in the profession. On Tuesday they held a meeting, of which the following minute has been furnished us:

At a meeting of the Physicians of Atlanta, held at the Young Men's Library, Tuesday morning, March 15, at 9 o'clock, Dr. T. J. Word was called to the chair, and Dr. E. van Goidtsnoven requested to act as secretary.

The chairman stated that the object of the meeting was to take some action in regard to the death of Dr. J. J. Caldwell, an old and esteemed medical brother of this city; whereupon a committee, consisting of the following persons, to wit, Dr. R. C. Word, Dr. E. L. Connally, Dr. K. C. Divine, Dr. E. J. Roach, Dr. J. Stainback Wilson, Dr. J. W. Duncan, and Dr. J. S. Todd, was appointed to draw up suitable resolutions expressive of the views and feelings of this meeting in regard to the sad event.

The committee, after brief cousultation, reported as foliows:

Whereas, Dr. J. J. Caldwell, our old and highly esteemed medlcal friend and brother, departed this life on Sunday last, the 14th of March, 1886, at his residence in this city; and whereas, he was eminent and beloved in the profession, an upright, consistent Christian man; therefore

Resolved, That his presence, as a citizen and friend, and his valuable services as a physician, will be greatly missed, and his departure deeply deplored by numerous friends in this community.

Resolved, That we extend to the immediate relatives and friends of the deceased our heartfelt sympathy and condolence in this their time of sore bereavement and sorrow.

Resolved, further, That these resolutions be published in the city papers, and that copies of the same be furnished the family of the deceased.

On motion, the above was unanimously adopted.

